# Iyengar Yoga versus Enhanced Usual Care on Blood Pressure in Patients with Prehypertension to Stage I Hypertension: a Randomized Controlled Trial

**DOI:** 10.1093/ecam/nep130

**Published:** 2011-02-14

**Authors:** Debbie L. Cohen, LeAnne T. Bloedon, Rand L. Rothman, John T. Farrar, Mary Lou Galantino, Sheri Volger, Christine Mayor, Phillipe O. Szapary, Raymond R. Townsend

**Affiliations:** ^1^Renal Division, University of Pennsylvania School of Medicine, Philadelphia, PA 19104, USA; ^2^Division of General Internal Medicine, University of Pennsylvania School of Medicine, Philadelphia, PA 19104, USA; ^3^Center for Clinical Epidemiology and Biostatistics, University of Pennsylvania, Philadelphia, PA 19104, USA; ^4^Program in Physical Therapy, Richard Stockton College of New Jersey, Pomona, NJ 08240, USA

## Abstract

The prevalence of prehypertension and Stage 1 hypertension continues to increase despite being amenable to non-pharmacologic interventions. Iyengar yoga (IY) has been purported to reduce blood pressure (BP) though evidence from randomized trials is lacking. We conducted a randomized controlled trial to assess the effects of 12 weeks of IY versus enhanced usual care (EUC) (based on individual dietary adjustment) on 24-h ambulatory BP in yoga-naïve adults with untreated prehypertension or Stage 1 hypertension. In total, 26 and 31 subjects in the IY and EUC arms, respectively, completed the study. There were no differences in BP between the groups at 6 and 12 weeks. In the EUC group, 24-h systolic BP (SBP), diastolic BP (DBP) and mean arterial pressure (MAP) significantly decreased by 5, 3 and 3 mmHg, respectively, from baseline at 6 weeks (*P* < .05), but were no longer significant at 12 weeks. In the IY group, 24 h SBP was reduced by 6 mmHg at 12 weeks compared to baseline (*P* = .05). 24 h DBP (*P* < .01) and MAP (*P* < .05) decreased significantly each by 5 mmHg. No differences were observed in catecholamine or cortisol metabolism to explain the decrease in BP in the IY group at 12 weeks. Twelve weeks of IY produces clinically meaningful improvements in 24 h SBP and DBP. Larger studies are needed to establish the long term efficacy, acceptability, utility and potential mechanisms of IY to control BP.

## 1. Introduction

Hypertension remains a major public health issue with the latest data from the National Health and Nutrition Examination Survey (NHANES) revealing that 65 million adults in USA have hypertension [1]. Hypertension is associated with increased risks of stroke, cardiovascular disease and chronic kidney disease [2]. Patients with high normal blood pressure (BP) [systolic BP (SBP) 130–139 mmHg or diastolic BP (DBP) 80–89 mmHg] fall into the category of prehypertensive [3] and are at an increased risk for adverse cardiovascular events compared with normotensive controls [4]. Lifestyle modifications (LSM) are recommended as first line approach for both prehypertensive and Stage 1 hypertension patients (SBP 140–159 mmHg or DBP 90–99 mmHg) [5]. LSM include weight loss, dietary recommendations and increased physical activity which can reduce SBP by 2–20 mmHg [3], but are often difficult to sustain [6].

There is emerging data that mind-body therapies (MBTs) may be useful in managing modest elevations in BP [7]. Yoga, a movement-based MBT, is especially attractive as a candidate therapy in the management of elevated BP because of its wide appeal to Westerners and its combination of gentle physical activity, slowed regulated breathing and meditation. If yoga practice were to be effective in preventing, delaying or treating mild hypertension, this could translate into a clinical and health economic benefit in people motivated enough to pursue this kind of treatment. There are several reports in the literature on the effects of various yoga programs on BP [8–16]; however, most of these are case series or studies with methodological limitations.

The objective of this randomized controlled trial was to rigorously evaluate the cardiovascular and physiologic effects of a 12-week structured Iyengar yoga (IY) program compared with an enhanced usual care (EUC) intervention emphasizing dietary approaches on reducing average SBP as measured by 24-h ambulatory BP monitoring (ABPM) in adults with untreated prehypertension to Stage I hypertension.

## 2. Methods

### 2.1. Participants

Participants included 78 yoga-naïve adults between the ages of 22 and 69 years with untreated SBP ≥130 mmHg but <160 mmHg, and DBP <100 mmHg. Major exclusion criteria included: pregnancy or postpartum <3 months; current use of any medications or dietary supplements known to affect BP; body mass index (BMI) >40 kg/m^2^; practice of IY in previous 12 months, or active practice of any other MBT more than two times per month; diabetes mellitus; cardiovascular disease; autonomic neuropathy; current tobacco use; renal insufficiency; and >10 alcoholic drinks per week in women and >15 drinks in men. Subjects were recruited by flyers placed in the hospital and university campus and by advertisements placed in local papers and online advertising using craigslist. The University of Pennsylvania Institutional Review Board approved the trial, and all subjects provided written, informed consent. The trial was registered at the Clinical Trials website: NCT00328666.

### 2.2. Study Protocol

Screening BP and heart rate (HR) were measured in the morning after a 12-h fast using a Datascope Accutorr Plus machine with an appropriate sized cuff after individuals were seated for 5 min. Three readings were obtained, separated by 1-min intervals and the average of these readings determined eligibility. Eligible subjects returned to the General Clinical Research Center (GCRC) for an inpatient stay at Weeks 0, 6 and 12 for ABPM which was recorded over 24 h using Spacelabs model 90207 monitors [17]. Subjects remained in the GCRC during the inpatient stay and were sedentary. Data was determined to be satisfactory if there were at least 48 (80%) acceptable readings (SBP between 70 and 280 mmHg and DBP between 40 and 150 mmHg) between 6 a.m. and 12 : 00 midnight, and six acceptable readings between midnight and 6 a.m. If any three consecutive readings averaged >180 mmHg for SBP, or >110 mmHg for DBP, the participant was discontinued.

### 2.3. Intervention

Over 23 months, seven cohorts containing 8–12 participants per cohort were recruited sequentially and randomized to either EUC or IY after completing the week 0 visit and prior to discharge from their first GCRC overnight stay. Participants randomized to IY attended twice weekly 70 min IY classes for the first 6 weeks (first class was within 7 days after week 0 visit) and then once a week for the following 6 weeks. Classes were not open to the public and were taught by two IY-certified instructors. In each class, 2–10 participants were led through a sequence of adjusting the body into timed postures (asanas) and breathing techniques (pranayama) using props. The IY program (Table 1) was developed by IY-certified instructor, Joan White, with input from IY founder B.K.S. Iyengar and included specific postures that were easy to adapt for older, obese populations. During the second-half of the program (Weeks 6–12), IY participants also participated in home practice using a 25-min DVD. Each participant kept a home diary to document frequency and duration of the home practice. Participants who missed three consecutive or four total IY studio classes were discontinued. Participants randomized to IY did not receive any instructions or guidance regarding LSM. 

Participants randomized to EUC classes attended four 1-h group classes that met during Weeks 1, 2, 3 and 8 with 30-min individual phone contact at Weeks 5 and 10. Classes were taught by the same registered dietitian (S.V.). The classes were designed to include motivational and behavioral components educating participants about LSM to reduce elevated BP including weight reduction/maintenance, sodium reduction, alcohol restriction and increasing intake of fruits, vegetables and low-fat, calcium-rich foods. EUC participants who missed more than one of the first three classes were discontinued.

At each visit, body weight was measured on a calibrated scale. Subjects were instructed to record all food and beverages consumed for three assigned days prior to Weeks 0, 6 and 12. Data was analyzed using Nutrition Data Systems for Research software, version 4.05, University of Minnesota.

In order to assess the effect of IY on the hypothalamic-adrenal axis, we measured salivary cortisol and several urinary and blood biomarkers. Caffeine, alcohol and exercise were discouraged 8 h prior to collection. Salivary cortisol samples were collected between 7 a.m. and 8 a.m. and sealed into a container until analyzed by an ELISA assay (Salimetrics). Urine was collected over 24 h at each visit and stored at 4°C during collection and transferred to a −70°C freezer until analyzed. Blood was obtained after a 12-h fast at Weeks 0, 6 and 12. Urine cortisol was measured after extracting with dichloromethane using a radioimmunoassay kit (Siemen Medical Solutions Diagnostic, Los Angeles, CA, USA) Urine sodium and potassium were measured using a Laboratory Instrument 943 flame photometer. Plasma Aldosterone was also measured using the radioimmunoassay kit (Siemen Medical Solutions Diagnostic). Plasma Renin was measured by IRMA using a kit by Diagnostic Systems Laboratories, Webster, TX, USA. Plasma Metanephrines were measured by ELISA using a kit by ImmunoBiological Laboratories, MN55432.

The following psychometric evaluations were performed at Weeks 0, 6 and 12 to determine the effect of IY on mood, anxiety, stress and health-related quality of life as compared with EUC. The specific tests performed included the Profile of Mood States (POMS), the Perceived Stress Survey (PSS) and the Short-form Health Survey (SF-36). Research staff manually scored the completed questionnaires and the results were entered into the database.

### 2.4. Statistics

#### 2.4.1. Sample Size

All sample size calculations were done and verified using Power and Precision version 2.04 (Dataxiom Software Inc.), which estimated that with a 15% dropout rate, we would need to randomize 30 subjects per group. The sample size was based on an 80% power calculation to detect a 4 mmHg SBP difference between groups.

#### 2.4.2. Statistical Analysis

Differences in baseline characteristics between subjects who completed and those who dropped out were tested using *t*-tests for continuous variables and chi-squared tests for categorical variables. Outcomes within the IY group and within the EUC group were tested by comparing means at baseline to means at Week 6 and means at Week 12 using *t*-tests on the equality of means. Outcomes for the IY group relative to the EUC group were tested by comparing the difference in means between baseline and Week 6 and between baseline and Week 12 for the IY arm and the EUC group. The standard *t*-test of differences in means assumes that the variances are equal. This assumption may be too strong. If violated, it may underestimate the true standard error and lead to *P*-values that are too small. We used Satterthwaite's formula to correct the standard error and *P*-values when needed.

## 3. Results

### 3.1. Study Participation and Retention

**Figure 1:**
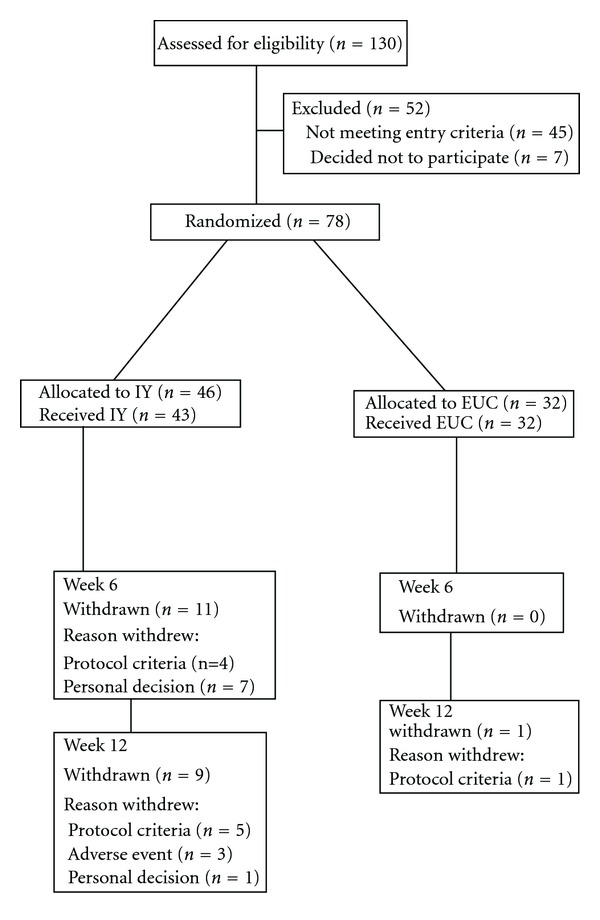
Study flow diagram.

Figure 1 shows participant retention. Dropout rate exceeded the estimated 15% in the IY arm. Because the dropout rate was imbalanced (zero in the EUC arm at this time), randomization was changed from 1 : 1 to 4 : 1 (IY to EUC) for the last cohort to try and balance group numbers.

### 3.2. Demographics

Baseline demographics of enrolled subjects are shown in Table 2. There were no significant baseline differences between those who dropped out or completed or between completers when comparing treatment groups. 

### 3.3. Twenty-Four Hour AMBP

**Figure 2:**
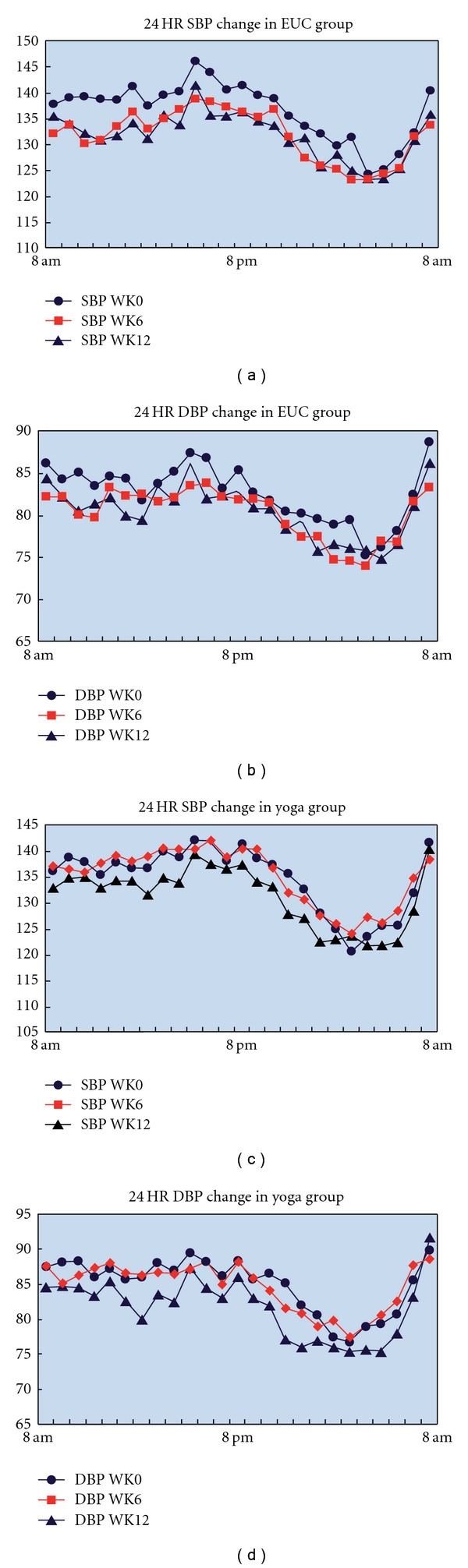
Changes in mean 24 h SBP and DBP in the IY and EUC groups at randomization, 6 and 12 weeks.

SBP and DBP of 24 h at Weeks 0, 6 and 12 are available from 26 people in the IY group and 31 people in the EUC group, which is shown in Table 3. At 6 weeks, 24 h SBP significantly improved in the EUC group when compared with the IY group (*P* value between groups = .04). There was no significant difference in 24 h DBP at 6 weeks between groups and no difference in either 24 h SBP or DBP at 12 weeks between groups. Among IY participants, there was no significant change in 24 h SBP or DBP at 6 weeks compared to baseline, but both measures improved at 12 weeks (*P* = .05 for SBP and *P* < .01 for DBP). Within the EUC group, 24 h SBP and DBP significantly decreased from baseline at 6 weeks (*P* = .02, SBP; *P* < .05, DBP), but changes in these measures at 12 weeks were no longer significant. This data is shown graphically in Figure 2. 

### 3.4. Weight and BMI

There was no significant change in weight or BMI over time between groups. However, weight and BMI decreased significantly in the EUC group at 12 weeks compared to baseline. Weight decreased from 88.8 ± 3 kg at Week 0 to 87.2 ± 0.7 kg at Week 12 (with a change of −1.6 kg, *P* = .02) in the EUC group. There was no change in weight in the IY group (85.5 ± 3.9 kg at Week 0 and 87.0 ± 1.8 kg at Week 12; change of 1.5 kg, *P* = 0.4). BMI also decreased significantly in the EUC group at 12 weeks (*P* < .05) but remained unchanged in the IY group at 12 weeks.

### 3.5. Diet Composition

The IY group did not receive any individualized dietary instructions or guidelines. There was a significant decrease in energy intake at 6 weeks in the EUC group compared to the IY group that was due to a decrease in total fat and carbohydrates. EUC participants also had a significant decrease in dietary sodium intake at 6 weeks compared to the IY group, yet sodium levels had started to revert towards baseline at 12 weeks. Urine sodium levels also showed a significant decrease at 6 weeks implying dietary compliance with a sodium restricted diet; however, at 12 weeks, urine sodium levels had increased and were no longer significant. At 12 weeks, the change in potassium intake was significantly greater in the EUC group when compared with the IY group.

### 3.6. Biochemical Measures

There were no differences over time between treatments in plasma levels of aldosterone, renin or metanephrines or in urinary or salivary cortisol concentrations. There were no significant changes in routine chemistry measures between groups at 12 weeks.

### 3.7. Psychometric Evaluations

There were no significant inter- or intra-group differences in psychometric data at 6 or 12 weeks for the POMS and PSS. However, the SF-36 health transition subscale was statistically significant for the EUC group at 6 weeks (*P* = .02) but was not sustained at 12 weeks. Correlations between psychometric data and the change in 24 h SBP were found in PSS (*P* = .03), POMS (*P* = 0.4) and Global scores (*P* = .001) in both groups. There was a correlation between the change in the total SF-36 and change in salivary cortisol (*P* = .02) for both groups (data not shown).

## 4. Discussion

This is the first randomized controlled clinical trial in USA assessing the effects of IY compared with dietary measures on 24 h ABPM in patients with prehypertension or Stage I hypertension. Although we did not find that IY significantly improved any measured endpoint over the EUC group, our data demonstrated that IY produced clinically meaningful reductions in SBP and DBP at 12 weeks compared to baseline within the IY group. The mean 24 h SBP decreased by 6 mmHg at 12 weeks compared to baseline. This is comparable to estimated reductions in SBP when following individual established LSM including weight loss, DASH diet, sodium reduction or physical activity [3] and was numerically greater than the change in mean 24 h SBP in the EUC group at 12 weeks. Data from a meta-analysis of individual data for 1 million adults from 61 prospective observational studies of BP and mortality demonstrated that even a small 2-mmHg fall in mean SBP was associated with a 7% lower risk of ischemic heart disease (IHD) death and a 10% lower risk of stroke death [18] emphasizing that even small reductions in SBP in large populations can have important cardiovascular morbidity and mortality benefits.

Psychological stress is a risk factor for hypertension [19–21] and the Canadian Hypertension Society recognizes the role of stress management techniques in managing hypertension [22, 23]. Reduction of stress using transcendental meditation has been shown to decrease BP in African American hypertensives [24, 25]. Various forms of yoga also claim to reduce stress by integrating the body and mind. Although there are several published studies investigating the effects of various forms of yoga on hypertension [8, 9, 12, 13, 26], most of these were uncontrolled case reports or small cohort studies conducted in India with significant methodological limitations [8, 11, 12]. There are only three randomized controlled trials of any form of yoga for hypertension [9, 10, 26]. The most recent study was an 8-week pranayama and asana yoga program conducted in 27 untreated hypertensive patients and 27 controls in Thailand [26]. The experimental group significantly reduced SBP by 24 mmHg at 8 weeks compared to 2 mmHg increase in the control group. DBP significantly improved by 18 mmHg in the experimental group compared to an increase of 2 mmHg in the control group. Details regarding methods and accuracy of BP measurement were not included. In India, 33 hypertensive adults were randomly assigned to three groups (yoga, medications only, or no therapy) and were followed for 11 weeks [9]. Yoga was performed at home for 6 h per week; at the end of the study, SBP reduced by an impressive 33 mmHg compared with 4 mmHg in the control group and 24 mmHg in the poorly described drug therapy group. The differences were significant compared with both control and drug treatment. In an older RCT from England, 43 patients with known hypertension, most of whom were already medically treated, were randomized to yoga plus biofeedback or usual care [10]. Treatment reduced SBP by 26 mmHg versus 9 mmHg in the control (*P* < .005). However, this study used a mixed intervention which included biofeedback in addition to yoga, which was more like TM and did not include any movement.

There are several limitations that can affect interpretation of the data. Subjects enrolled in the trial were possibly more likely than the general population to be motivated to pursue a lifestyle modification such as yoga as a therapy for hypertension and this can lead to a self-selected study population being enrolled in the trial. Although randomized, the trial was unblinded and though we included the EUC control group, it was an active control. While blinding is ideal to eliminate measurement bias, it is not feasible with behavioral interventions. The EUC intervention produced significant changes in some BP parameters and although numerically smaller than the IY group, at 12 weeks we were underpowered to detect a statistically significant difference between groups. The lack of differences between groups may be attributed to both the unanticipated high dropout rate in the IY group and the duration of the trial which was probably too short to detect greater changes in the IY group as it is apparent that changes in BP in the IY group took longer to occur.

The EUC group produced a significant reduction in 24 h SBP at 6 weeks compared to the IY group as well as a significant within group improvement in DBP. While these variables were not statistically significant at 12 weeks either between groups or within the EUC group, they were still clinically meaningful and met the primary aim of decreasing SBP by 6 mmHg from baseline. Changes in 24 h SBP and DBP were comparable to changes seen in previous clinical trials assessing the effects of dietary modifications alone [27, 28] and in combination with other lifestyle modifications on BP [29]. Body weight in EUC-randomized subjects decreased significantly within this group at 12 weeks compared to baseline. EUC participants made significant improvements in energy, fat, carbohydrates and sodium intake at 6 weeks, yet all variables started to revert towards baseline at 12 weeks. This was seen particularly with urine sodium which showed a significant decrease at 6 weeks implying dietary compliance with a sodium-restricted diet; however, at 12 weeks, urine sodium levels had increased and were no longer significant indicating difficulty in maintaining dietary sodium restriction. These findings are supported by others revealing that dietary recommendations are hard to follow over time [6].

Another limitation is that the two intervention groups met with unequal frequency and this can introduce confounding. Due to the high attrition rate in the IY group which exceeded expectations, a change was necessitated in the randomization scheme for the last cohort from 1 : 1 to 4 : 1 (IY: EUC). Although we acknowledge this as a limitation, there were no differences in the baseline demographics in the IY group in the subjects who dropped out compared to the subjects who completed. Twenty participants (43%) randomized to IY did not complete, thereby reducing the numbers of completers and thus the power of the trial. Of the IY non completers, 60% were due to not being able to adhere to the class schedule. This may be due to several reasons. IY is not an aerobic form of exercise like other more Westernized forms of yoga, and subjects recruited into the study were “yoga-naïve" and may have expected a more traditional type of yoga and may have been discouraged by the rigid style of our IY program. The yoga program was also designed as “top heavy" program which decreased in intensity over the trial. Yoga practice for naïve yoga subjects requires slow transition with increase in intensity of the yoga program over a few months.

We sought to determine the role of reductions in stress as a mechanism for the BP pressure changes in our subjects using questionnaires and salivary cortisol. Our failure to find such association in the IY group was disappointing. Other studies evaluating yoga practice suggests that yoga may reduce oxidative stress [30] and endothelial-dependent vasodilatation in patients with established coronary artery disease [16]. Additional studies are needed incorporating measures of oxidative stress and endothelial function.

Our data demonstrates that the effects of IY on BP at 12 weeks as assessed by 24 h ABPM were comparable or greater than that produced by an EUC intervention in adults with prehypertension and stage 1 hypertension. This pilot has provided useful data and experience that we can use for future studies. Specifically, when designing a future trial, we would include three groups and assess the combination of EUC and yoga versus intervention alone, we would enroll subjects for a longer duration (24 weeks) to detect greater, more sustained reductions in BP in the yoga groups. We would redesign the yoga program with specific attention to improving adherence and gradually increasing yoga practice over the course of the trial. We would also consider using a less rigid, more aerobic type of yoga that is more available and accessible to the public and therefore more likely to be incorporated into patients' lifestyles as a long-term lifestyle adjustment. However, given the general safety profile of yoga and general public appeal, we feel it is important to fund additional studies in order to establish the long-term efficacy and the potential mechanisms in BP reductions associated with yoga therapy in patients with prehypertension and Stage 1 hypertension.

## Funding

National Institutes of Health grants R21AT002353-02 (to P.O.S. and R.R.T.) and M01-RR00040 (General Clinical Research Center) and National Center for Complementary and Alternative Medicine (NCCAM).

## Figures and Tables

**Table 1:** IY class structure and components.

Component ^(a)^	Time
Instructor introduction and organizational issues	5 min
Set up and take attendance	5 min
Asanas ^(a)^	
Savasana (corpse pose)	5 min
Cross bolsters	5 min
Supta baddha konasana (supine bound angle pose) ^(a)^	5 min
Supta swastikasana (supine fortunate pose) ^(a)^	5 min/side
Bharadvajasana (a twisting pose)	3 × 30 s/side
Pavannamuktasana (release of wind pose)	5 min
Adho mukha virasana (downward facing hero pose)	5 min
Adho mukha swastikasana (downward facing fortunate pose) ^(a)^	1 min/side
Adho mukha svanasana (downward facing dog)	1 min
Uttanasana (standing forward bend)	1 min
Janu sirsasana (seated forward bent with bent leg)	1 min/side
Upavisthakonasana (seated forward bend with wide legs)	3 min
Paschimottanasana (full forward bend) ^(a)^	1 min
Savasana (corpse pose) ^(a)^	5 min
Ujjayi (the conqueror) pranayama ^(a)^	5 min
End of class attendance ^(a)^	
Total class time (including 1 min transitions) ^(a)^	*∼*71 min

^(a)^Included in the home program.

**Table 2:** Baseline demographics by treatment ^(a)^.

Characteristics	IY	EUC
	(*n* = 46)	(*n* = 32)
Male, *n*	23	16
Female, *n*	23	16
Race, *n*		
African American	18	14
Asian/native Hawaiian/Pacific Islander	3	1
Caucasian	21	16
Hispanic	3	0
Other	1	1
Age (years)	48.2 ± 1.6	48.3 ± 2.4
Weight (kg)	86.4 ± 3.1	87.7 ± 3.4
BMI (kg/m^2^)	29.6 ± 0.9	30.5 ± 1.0
SBP (mmHg)	140 ± 1	140 ± 1
DBP (mmHg)	87 ± 1	86 ± 1

^(a)^Results reported as mean (standard error).

**Table 3:** Changes in 24 h BP parameters ^(a)^.

	IY			EUC		
	Week 0	Week 6	Week 12	Week 0	Week 6	Week 12
SBP (mmHg)	132 (3)	133 (2)	126 (3)*	135 (3)	130 (2)**	131 (2)
DBP (mmHg)	83 (2)	83 (1)	78 (2)***	82 (2)	79 (1)**	80 (1)
MAP (mmHg)	99 (2)	99 (1)	94 (2)**	99 (2)	96 (1)**	97 (2)
Heart rate (mmHg)	70 (2)	70 (1)	68 (2)	69 (2)	67 (1)	67 (1)

^(a)^Results reported as mean (standard error).

*P = .05, **P < .05, ***P < .01.
